# Primary mucinous sarcoma of the lung with EWSR1 translocation: a case report

**DOI:** 10.3389/fmed.2025.1704915

**Published:** 2025-12-04

**Authors:** Chuchu Xu, Xiaona Yin, Xi Wang, Yongsheng Wang, Xiaoqiong Wang

**Affiliations:** The Second People’s Hospital of Hefei, Hefei Hospital Affiliated to Anhui Medical University, Hefei, Anhui, China

**Keywords:** primary mucinous sarcoma of the lung, EWSR1 gene translocation, diagnostic imaging, computed tomography, bronchoscopy

## Abstract

Primary pulmonary myxoid sarcoma (PPMS) is an extremely rare lung sarcoma with a low incidence. It is a low-grade malignant tumor with histological and molecular features similar to those of extranodal myxoid chondrosarcoma. To date, it has been reported less frequently. We report a case of primary mucinous sarcoma of the lung with EWSR1 translocation, which was definitively diagnosed by bronchoscopic biopsy. Biopsy tissues were tested for pathology, immunohistochemistry, and molecular pathology. Additionally, fluorescence *in situ* hybridisation was performed using an EWSR1 breakthrough probe. The patient’s left common branch was obstructed by a neoplasm, which was identified during bronchoscopy. A tracheoscopic circling procedure was then performed. The patient underwent a small-incision sleeve resection of the lower lobe of the left lung and lymph node dissection with the assistance of video-assisted thoracic surgery (VATS) under general anesthesia. The patient had a good postoperative recovery, and a follow-up chest CT at 2 months after the operation showed no signs of recurrence.

## Introduction

In 1999, Nicholson et al. first reported two cases of what was then classified as a new type of low-grade malignant mucinous endobronchial tumor (1). Subsequently, in 2011, Thway et al. introduced the term PPMS based on its genetic signature EWSR1-CREB1 (2). In the 2021 WHO classification of thoracic tumors, this tumor is designated as ‘PPMS with EWSR1-CREB1 fusion (3). PPMS is prevalent in young women and most of the cases are characterized by the presence of the gene EWSR1-CREB1 fusion in most of the cases (4). It has been found that PPMS typically exhibits a characteristic t(2;22)(q33;q12) chromosomal translocation resulting in an oncogenic fusion gene, EWSR1-CREB1 fusion. PPMS originates from mesenchymal components of the bronchial wall, lung stroma, or vasculature (5), and its clinical manifestations are nonspecific. We report a case of PPMS presenting with fever and chest tightness.

## Case history/examination

**Figure 1:**
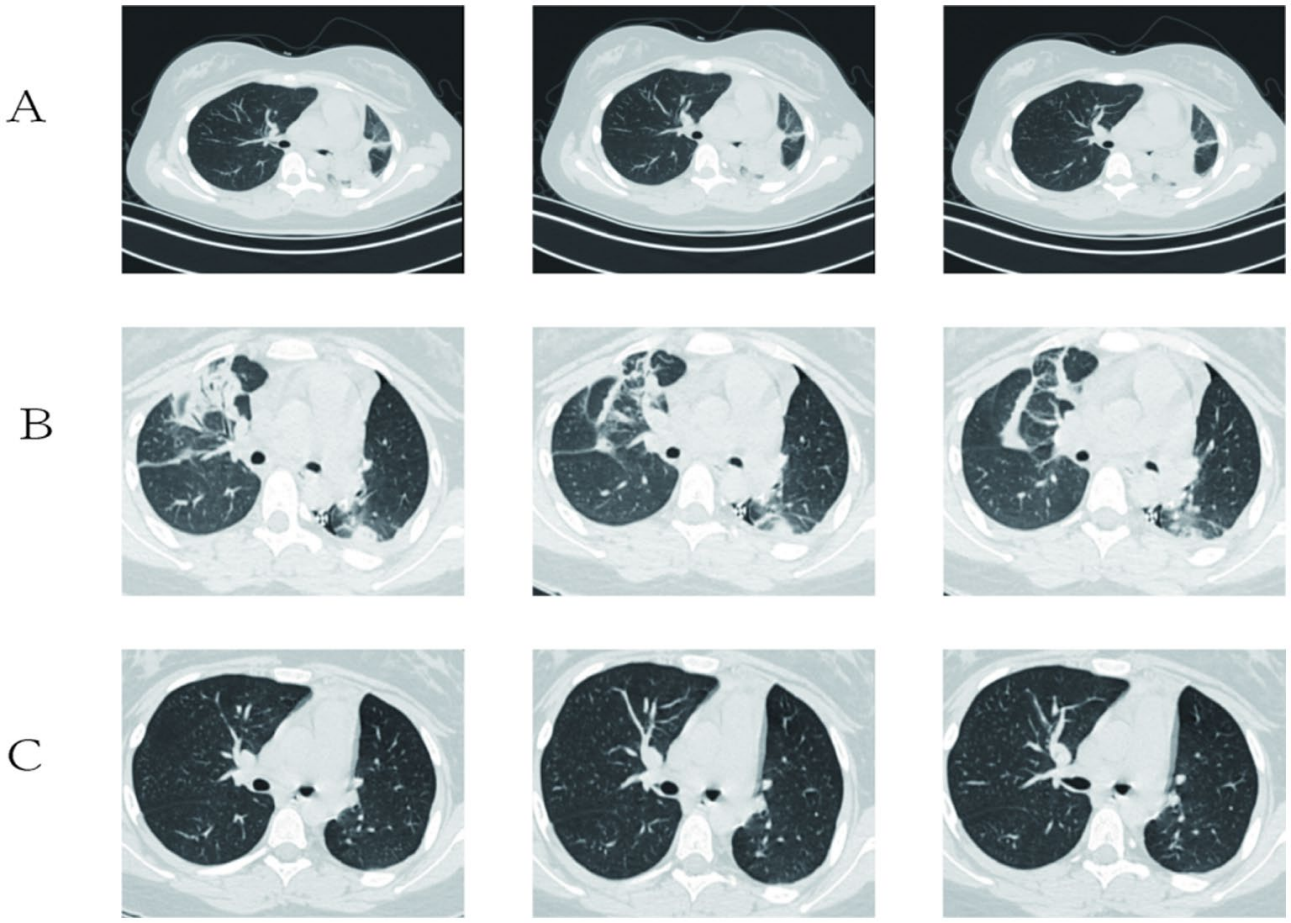
**(A)** CT scan revealing soft tissue shadows in the left main bronchus and scattered patchy hyperdense shadows in the left lung; **(B)** A chest CT taken 2 days after surgery displays striated solid shadows and multiple ground-glass patchy shadows in the upper and lower lobes of the left lung, with the lower lobe being the most prominent; **(C)** Repeat chest CT more than 2 months after the surgery did not show any obvious recurrence or metastasis.

A 14-year-old Han Chinese female student, with no reported significant family history of tumors or genetic syndromes, presented to a local hospital with fever and chest tightness. The patient has no history of smoking or drinking, no history of surgery, and no history of chronic diseases. The patient’s parents, grandparents, maternal grandparents, and siblings have no history of hereditary diseases. A chest CT scan indicated lung shadows. The patient did not report any cough, sputum production, or chest pain. A chest enhancement CT performed on April 10, 2024, revealed a soft tissue shadow in the left main bronchus, which was significantly enhanced post-contrast with a CT value ranging from 19 to 60 HU. Distal patches of increased density with fuzzy borders and an air bronchogram sign were observed. The mediastinum was shifted to the left. Scattered patchy hyperdense shadows with blurred borders were noted in the left lung. No obvious enlargement of lymph nodes was observed in the pulmonary hilum or mediastinum. There was no significant pleural thickening on either side, and a small amount of fluid was present in the left pleural cavity (Figure 1A).

**Figure 2:**
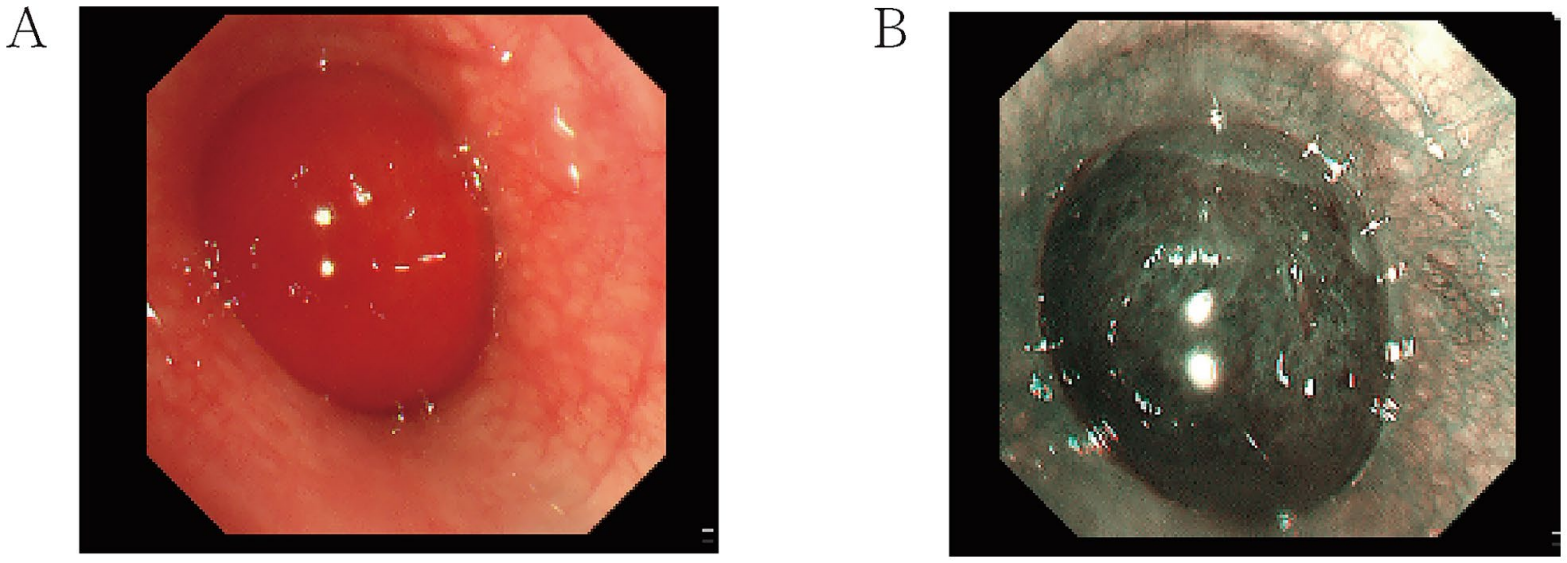
**(A)** Tracheoscopy showed that the left common branch neoplasm obstructed the lumen. The distal lumen was not visible, and the surface of the neoplasm was smooth and rich in blood supply. **(B)** NBI showed that the vascularity of the distal neoplasm of the left common branch was tortuous and thickened.

**Figure 3:**
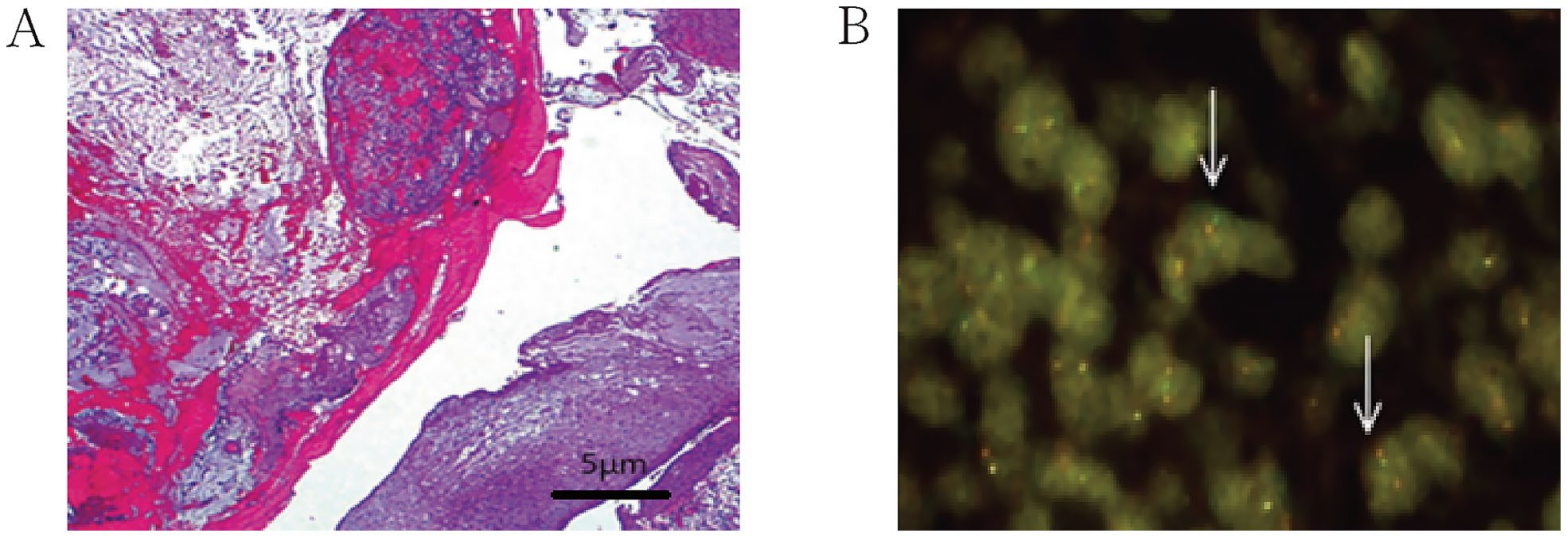
**(A)** Oval and short spindle cell proliferative lesions were observed (HE ×100); **(B)** The EWSR1 breakage probe was used for fluorescence *in situ* hybridization, revealing a separation of red and green signals (arrows), indicating EWSR1 translocation.

Primary pulmonary mucinous sarcoma is a rare type of lung tumor classified as a sarcoma. Its differential diagnosis primarily includes the following conditions: Lung adenocarcinoma: Lung adenocarcinoma is one of the most common primary lung cancers, typically presenting as lung nodules or masses. A definitive diagnosis requires tissue biopsy. Adenoid cystic carcinoma: This is a rare type of lung tumor that may resemble myxoid sarcoma in appearance. Immunohistochemistry and genetic testing are often needed to distinguish between the two. Lung metastases: Metastases from tumors in other parts of the body, particularly adenocarcinoma and breast cancer, may form similar lesions in the lungs and require differentiation on imaging studies. Sarcomas: Such as leiomyosarcoma and osteosarcoma, although rare, may overlap with myxoid sarcoma on imaging. Benign tumors: Such as pulmonary hamartomas and pulmonary cysts, typically have characteristic imaging features, and a diagnosis can be made based on clinical presentation and imaging results. Tracheoscopy revealed that the left main bronchial neoplasm obstructed the lumen with good mobility. The distal lumen was not visible. The surface of the neoplasm was smooth and richly supplied with blood (Figure 2A). Narrow-band imaging showed that the blood vessels of the distal left main bronchial neoplasm were tortuous and thickened (Figure 2B). No bleeding was observed during puncture. After the lesion was removed using bronchoscopic clamping and electrocautery, the lumens of the left upper lobe and left lower lobe were clear. The tip of the tumor was located at the opening of the left upper lobe. Histopathology of the biopsy from the left main bronchial mass suggested ovoid and short spindle cell proliferative lesions, with a potential for malignancy (Figure 3A). Further refinement of immunohistochemistry and molecular pathology revealed the following results: CD56 (locally positive), P40 (negative), Napsin A (negative), CK (negative), TTF-1 (negative), KI-67 (hotspot 5–10% positive), EMA (negative), SMA (negative), CD34 (negative), S-100 (focally positive), ERG (negative), VIM (positive), DES (partially positive), ER (negative), PR (negative), MDM2 (negative), ALK (D5F3) (negative), MyoD1 (negative), Myogenin (negative). Fluorescence in-situ hybridization using the EWSR1 breakage probe showed a separation of the red and green signals (arrows), suggestive of EWSR1 translocation, with EWSR1 gene split signal-positive cells accounting for about 20% of the total number of tumor cells (Figure 3B). Due to the limited number of pathological specimens and technical constraints, this study was unable to complete molecular testing for EWSR1 fusion partners, which constitutes a clear limitation. Based on morphological and molecular findings, primary mucinous sarcoma of the lung with EWSR1 translocation was diagnosed. On May 7, 2024, a VATS-assisted small-incision sleeve resection of the lower lobe of the left lung, combined with lymph node dissection, was performed under general anesthesia. Chest CT was repeated 2 days after the operation (Figure 1B): the left thoracic cavity was narrower, with a deviated mediastinum and an upwardly displaced diaphragm. Striated solid shadows and multiple ground glass patchy shadows were seen in the upper and lower lobes of the left lung, with the lower lobe being prominent. Patchy and solid metaplasia was seen in the upper and middle lobes of the right lung, and a few patchy and striated micronodular shadows were seen in both lungs. There were no obvious enlarged lymph nodes in the mediastinum. Small amount of pericardial effusion. The left thoracic drainage was in progress. Subcutaneous pneumoperitoneum in the left chest wall. Symptoms resolved after symptomatic treatment and he was discharged from the hospital, and no obvious recurrence or metastasis was found on the chest CT on review more than 2 months after the operation (Figure 1C). One year later, the patient’s family was contacted again for a routine follow-up examination, which revealed no recurrence of the tumor. We have thoroughly communicated with the patient’s family regarding the necessity and potential scientific significance of the RT-PCR test and sought their informed consent. After careful consideration, the patient’s family has currently declined to arrange for additional blood sample collection for this test. We respect the family’s right to make their own decisions, and therefore cannot proceed with this supplementary testing at this stage.

## Discussion and conclusion

PPMS, a rare, low-grade malignant sarcoma associated with EWSR1 gene translocation, has a low incidence, and its histological origin and biological behavior remain unknown. The tumor often presents endobronchially and may infiltrate the surrounding lung parenchyma. PPMS is a recently described lung sarcoma more prevalent in young females with a characteristic genetic EWSR1-CREB1 fusion in most cases (6). The majority of cases were confirmed to harbor the EWSR1-CREB1 gene fusion via RT-PCR, FISH or next-generation sequencing techniques. PPMS is characterized by an extensive mucinous stroma and a multilobular structure containing spindle or stellate-to-polygonal tumor cells arranged in a rope-like or reticular pattern with mild to moderate heterogeneity (7). The EWSR1 fusion gene is also observed in tumors such as angiomatoid fibrous histiocytoma and clear cell sarcoma, but PPMS is morphologically distinct from these tumors (8).

PPMS is an exceptionally rare, low-grade sarcoma. Patients exhibit no marked gender predilection, with clinical manifestations presenting diverse patterns. Coughing is one of the common symptoms in patients with PPMS. Some individuals may experience haemoptysis, which generally indicates more severe pathology or concomitant pulmonary conditions. This symptom tends to worsen progressively with disease advancement, while others may develop shortness of breath or wheezing. In certain cases, pulmonary masses may be incidentally detected during physical examinations or investigations for other conditions, without any apparent clinical symptoms. This retrospective review systematically collates and analyzes literature on primary progressive multiple myeloma with EWSR1::CREB1 fusion gene, examining its clinical, radiological, histological, and molecular characteristics alongside current therapeutic approaches (Table 1) (9).

**Table 1:** Reported cases of PPMS.

Case	Age (years)/Sex	Smoking history	Presentation	Site/Tumor size (cm)	Adjacent to bronchus	FISH result	RT-PCR result treatment follow-up (years)		
1	27/F	Never	NS	RLL/4	+	NR	NR	Surgery	NED/3
2	43/F	20 years	Bronchitis	L/13	+	NR	NR	Surgery	NED/0.5
3	60/M	NR	Asthma	R/9	−	NR	NR	Surgery	NED/3.8
4	27/F	Ex	NS	RLL/4	−	EWSR1 gene rearrangement	EWSR1-CREB1 fusion	Surgery	NED/15
5	33/F	Current	Cough	LUL/3.5	+	EWSR1 gene rearrangement	EWSR1-CREB1 fusion	Surgery	NED/12
6	45/F	NR	Cough	RUL/1.5	+	EWSR1 gene rearrangement	Neg	Surgery	NED/1
7	36/F	NR	Neural symptom	L/NR	NR	Neg	Neg	Surgery	Death followed a few months after the brain metastases
8	32/F	NR	Weight loss	RUL/NR	+	EWSR1 gene rearrangement	EWSR1-CREB1 fusion	Surgery	NR
9	28/M	Never	Cough, fever hemop- tysis, Weight loss	LLL/2.8	+	Neg	EWSR1-CREB1 fusion	Surgery	Left renal metastasis, alive/3
10	67/M	Current	NR	LLL/2.8	+	EWSR1 gene rearrangement	EWSR1-CREB1 fusion	Surgery	NR
11	68/F	NR	NR	RUL/2.0	+	Neg	Neg	Surgery	NR
12	63/F	Ex	hemoptysis	LUL/NR	+	EWSR1 gene rearrangement	EWSR1-CREB1 fusion	Surgery	NED/4
13	51/M	NR	HIV^+^	RLL/2.0	NR	EWSR1 gene rearrangement	EWSR1-CREB1 fusion	Surgery	NR
14	31/M	Never	NS	LLL/2.7	+	NR	EWSR1-CREB1 fusion	Surgery	NED/5.8
15	66/F	NR	Obstruction of the lung	LUL/4.0	+	EWSR1 gene rearrangement	EWSR1-CREB1 fusion	Surgery	NED/1.5
16	28/M	NR	Cough, hemoptysis	RLL/8.5	NR	EWSR1 gene rearrangement	Neg	Surgery	NED/1.3
17	28/M	NR	Stethalgia	RUL/6.0	+	Neg	Neg	Surgery	NED/0.3
18	26/M	NR	Cough, hemoptysis	LLL/9	+	EWSR1 gene rearrangement	EWSR1-CREB1 fusion	Surgery	NED/0.7
19	49/F	Never	NS	RLL/4	+	EWSR1 gene rearrangement	EWSR1-CREB1 fusion	Surgery	NED/9.7
20	54/F	Never	NS	RLL/4.5	+	EWSR1 gene rearrangement	EWSR1-CREB1 fusion	Surgery	NED/12.6
21	65/M	Never	Cough, stethalgia, expectoration	LLL/13	+	EWSR1 gene rearrangement	EWSR1-CREB1 fusion	Surgery	Metastases to lung/0.5, alive/6
22	29/F	NR	NS	L/3	−	EWSR1 gene rearrangement	EWSR1-CREB1 fusion	Surgery	NED/1.4
23	32/F	Never	Cough, Weight loss	RUL/3.5	+	EWSR1 gene rearrangement	EWSR1-CREB1 fusion	Surgery	NED/8
24	80/F	NR	tussiculation	LLL/NR	+	EWSR1 gene rearrangement	NR	Surgery	NED/3
25	48/M	Current	Cough	L/>14	+	Neg	Neg	Surgery	Metastases to Cerebellar/1, alive/1.7
26	41/F	NR	Stethalgia, dyspnea	R/5.1	+	EWSR1 gene rearrangement	EWSR1-CREB1 fusion	Surgery	NED/0.9
27	45/F	Never	NS	RUL/2.1	−	EWSR1 gene rearrangement	EWSR1-CREB1 fusion	Surgery	NED/3.1
28	24/M	Never	NS	RLL/5	−	EWSR1 gene rearrangement	NR	Surgery	NED/0.5
29	64/F	Never	Cough	RUL/5.5	+	EWSR1 gene rearrangement	NR	Surgery	Metastases to pleural/0.8; Metasta- ses to bone/1.1, alive/2
30	27/M	Current	Cough, hemoptysis	RLL/5.0	+	EWSR1 gene rearrangement	NR	Surgery	NED/2.4
31	45/M	Current	Cough	LLL/<3.0	+	EWSR1 gene rearrangement	NR	Surgery	NED/1.9
32	43/M	Current	Cough	RLL/2.0	+	EWSR1 gene rearrangement	NR	Surgery	NED/2.4
33	23/M	Current	Cough, fever	RLL/<3.0	+	EWSR1 gene rearrangement	NR	Surgery	NED/2
34	45/F	Never	Cough	RUL/2.0	+	EWSR1 gene rearrangement	NR	Surgery	NED/0.3
35	49/M	20 years	Cough, blood-stained sputum	R/14	+	NR	EWSR1-CREB1 fusion	Surgery	NED/0.1
36	47/M	NR	NS	RUL/2.0	+	EWSR1 gene rearrangement	NR	Surgery	NED/0.25
37 (Present case)	14/F	Never	NS	L/2.0	NR	EWSR1 gene rearrangement	NR	Surgery	NED/1

F, female; M, male; NS, No symptom; NR, not reported; NED, no evidence of disease; RUL, Right upper lobe; RLL, Right lower lobe; LLL, Left lower lobe; LUL, Left upper lobe; L, Left lung; R, Right lung; Adjacent to bronchus, endobronchial component involved (+) or not involved (−); Neg indicates negative no EWSR1 gene rearrangement or EWSR1 gene rearrangement.

PPMS is usually a solitary, well-demarcated mass with a lobulated appearance and a pale crystalline appearance on section. The size of the tumor varies from 1.4 cm to 14 cm, with an average size of 5 cm. During bronchoscopy, the tumor in this patient was found to be closely associated with the bronchus, displaying clear boundaries and a smooth surface. Imaging studies revealed lobulated soft tissue masses or cystic solid masses in the lungs with distinct boundaries, uneven density, and a close proximity to the bronchus. Additionally, the patient exhibited significant enhancement on CT imaging with a CT value ranging from 19 to 60 HU. It is important to note that PPMS lacks highly specific immunohistochemical markers. PPMS lacks highly specific immunohistochemical markers. Immunohistochemical analysis showed usually positive waveform proteins (9). Epithelial cell membrane antigens and CD99 may show focal or weak positivity, whilst other epithelial-derived markers, myogenic markers, and neurogenic markers are all negative. They did not exhibit cytokeratin (CK), thyroid transcription factor-1 (TTF-1), napsin A, S-100 protein, CAM5.2, CD10, CD31, CD34, desmin, smooth muscle actin (SMA), p63, calponin, h-caldesmon, interstitial lymphoma kinase (ALK), c-kit, synaptophysin, or glioblastic acidic protein (GFG), metaplastic lymphoma kinase (ALK), c-kit, melanocyte marker (HMB-45), synaptophysin, or glial fibrillary acidic protein (GFAP) immunoreactivity (10). Vimentin is an intermediate filament protein widely expressed in mesenchymal cells and commonly used for their labeling. It demonstrates positive expression across multiple tumor types, particularly in mesenchymal tumors, aiding in the differentiation of primary pleural mesothelioma (PPMS) from other non-mesenchymal tumors. Microscopically, a basophilic mucus-like stroma is often present, characterised in most cases by a multilobulated structure arranged in a striated, reticular or beam-like structure, and in the majority of cases there is a patchy inflammatory cellular infiltrate in the background, consisting mainly of lymphocytes and plasma cells.

Currently, data on chemotherapy for PPMS remain limited, with its efficacy yet to be definitively established, necessitating further investigation. Garnier et al. (11) initially reported a doxorubicin-based chemotherapy regimen for treating intracranial non-mucinous angioendothelioma employing the EWSR1::CREB1 transcript fusion, which maintained disease stability for 14 months after discontinuation. Subbiah et al. (12) suggested that patients harboring this EWSR1::CREB1 fusion may also benefit from crizotinib and pazopanib. Furthermore, Ngo et al. (13) reported a case of metastatic angiofibromatosis with EWSR1::CREB1 fusion and ALK overexpression demonstrating a durable response to crizotinib. However, the efficacy of crizotinib and pazopanib in treating PPMS with EWSR1::CREB1 fusion remains unexplored in clinical settings. Further data are needed to determine the suitability of these agents for treating PPMS with this specific fusion. Surgical resection is usually the mainstay of treatment for all patients with PPMS (10). PPMS, as a well-defined low-grade malignant solid sarcoma with a low Ki-67 index, has a good clinical prognosis after surgical resection (9). Based on the available literature, surgery is the primary treatment for most patients. Surgical resection methods include wedge resection, segmental resection, lobectomy, and total lung resection, with the choice dependent on the tumor’s size and stage. In this case, the patient underwent a VATS-assisted small-incision sleeve resection of the left lower lobe combined with lymph node dissection. No signs of recurrence or metastasis were observed during the 2-month follow-up period. One year later, the patient’s family was contacted again for a routine follow-up examination, which revealed no recurrence of the tumor. A young woman with fever and chest tightness underwent chest CT, revealing a lung metastasis. Immunohistochemical molecular pathology indicated that the cells were positive for EWSR1 gene split signals (accounting for about 20% of the tumor cells), suggesting a translocation of the EWSR1 gene. The histological characteristics of this case were similar to those reported in the literature, featuring ovoid and short spindle cell proliferative lesions. After resection of the lesion via bronchoscopy, a sleeve resection specimen of the left lower lobe was obtained. Examination revealed an absence of mucosal epithelium and evident extrusion wounds in parts of the bronchus. Additionally, the mucous membrane was mildly edematous, with submucosal fibrous tissue proliferation, mild proliferation of small blood vessels, and lymphocyte infiltration. No residual tumor was observed. Postoperative follow-up showed no recurrence of the lesion, suggesting a slow tumor progression and indicating that long-term prognosis requires further monitoring.

Due to the rarity and molecular heterogeneity of PPMS, standardized diagnostic and therapeutic approaches are currently lacking. Most cases rely on the detection of EWSR1 gene rearrangements, but this test does not provide a completely specific diagnosis for PPMS. Establishing more standardized diagnostic workflows and treatment protocols is needed in the future to enhance diagnostic accuracy and therapeutic efficacy. PPMS is a rare tumor that progresses slowly and has an unknown origin. It lacks clinical specificity and specific immunohistochemical markers, and is often associated with an EWSR1 translocation. The main treatment for PPMS is surgical resection, which typically results in a good prognosis.

## Data Availability

The original contributions presented in the study are included in the article/supplementary material, further inquiries can be directed to the corresponding authors.
